# Efficacy and Safety of Drug and Device Strategies for Stroke Prevention in Atrial Fibrillation After Intracranial Hemorrhage: A Bayesian Network Meta-Analysis

**DOI:** 10.3390/jcdd12120464

**Published:** 2025-11-28

**Authors:** Fenglin Qi, Yuhang Yang, Lili Wang, Sixian Weng, Qinchao Wu, Yijie Liu, Zhipeng Hu, Liying Chen, Yunlong Wang

**Affiliations:** Department of Cardiology, Beijing Anzhen Hospital, Capital Medical University, Beijing 100029, China; claire7766@mail.ccmu.edu.cn (F.Q.); yangyuhang193@163.com (Y.Y.); wang_lili1002@163.com (L.W.); wengzizhi@163.com (S.W.); wuqinchao1628@126.com (Q.W.); saintmikeliu@pku.edu.cn (Y.L.); huzhipeng976@163.com (Z.H.)

**Keywords:** atrial fibrillation, intracranial hemorrhage, anticoagulation, left atrial appendage occlusion, antiplatelet

## Abstract

(1) Background: Whether anticoagulation can be resumed in atrial fibrillation (AF) combined with intracranial hemorrhage (ICH), and which anticoagulation modality is used with better efficacy and safety, is unknown. (2) Method: Randomized controlled trials (RCTs) and observational studies on relevant topics were included by searching five databases: PubMed, EMBASE, EBSCO, Cochrane Central Register of Controlled Trial and ClinicalTrials. Bayesian network meta-analysis was performed to analyze the effect of oral anticoagulant (OAC), new oral anticoagulant (NOAC), warfarin, antiplatelet, left atrial appendage occlusion (LAAO) and no therapy in patients with AF after intracranial hemorrhage. (3) Results: We included 16 studies involving 25,483 patients. Compared with no antithrombotic therapy, the risk of thromboembolism and all-cause mortality were both reduced with OAC (OR: 0.38, 95% CI: 0.21–0.67; OR: 0.45, 95% CI: 0.25–0.8) and LAAO (OR: 0.11, 95% CI: 0.01–0.76; OR: 0.11, 95% CI: 0.01–0.88), and there was no increased risk of recurrent intracranial hemorrhage. Regarding thromboembolism, OAC (OR: 0.28, 95% CI: 0.11–0.69) was superior to antiplatelet therapy, and antiplatelet therapy (OR: 12.59, 95% CI: 1.57–133.50) was associated with a higher risk of thromboembolism than LAAO. There were no significant differences in recurrent intracranial hemorrhage between the interventions. LAAO appeared to be the best option for reducing thromboembolism (SUCRA: 0.96), recurrent intracranial hemorrhage (SUCRA: 0.75) and all-cause mortality (SUCRA: 0.94). (4) Conclusions: Based on this network meta-analysis, we hypothesize that LAAO has the highest likelihood of reducing the risk of thromboembolism and recurrent intracranial hemorrhage, as well as all-cause mortality in patients with AF after intracranial hemorrhage, followed by OAC.

## 1. Introduction

Atrial fibrillation (AF) is the most common sustained arrhythmia, increasing the risk of ischemic stroke (IS) by fivefold [1]. Anticoagulation is the core treatment for the prevention of IS and thromboembolism in patients with AF. Although anticoagulation is effective in reducing the risk of stroke and embolism in patients with AF, there are significant bleeding complications [2]. Among them, the most severe anticoagulation complication is intracranial hemorrhage (ICH).

Oral anticoagulation (OAC) therapy is the basis of anticoagulation in AF, including warfarin and NOAC. The use of OAC significantly reduces the risk of IS and thromboembolism in patients whose bleeding events also improve with the use of NOAC [3,4,5,6]. Left atrial appendage occlusion (LAAO) is a non-pharmacological modality for preventing IS and thromboembolic events in patients with AF, especially those with a history of significant bleeding and contraindications to OAC [2]. It should be noted that most of these studies of anticoagulation therapy excluded patients with AF combined with intracranial hemorrhage, so it is still being determined whether anticoagulation can be resumed in patients with AF combined with intracranial hemorrhage and how anticoagulation strategies should be chosen.

Previously, some studies have conducted preliminary explorations on whether to resume anticoagulation and anticoagulation strategies in patients with AF after intracranial hemorrhage, including warfarin, NOAC anticoagulation, antiplatelet therapy or LAAO. However, most of these studies have been limited to comparisons of a single anticoagulation modality versus cessation of anticoagulation or direct comparisons of two anticoagulants and have been affected by the influence of different populations and regions on the choice of treatment [7,8,9,10,11], resulting in a lack of evidence for clinicians to treat such patients.

Therefore, we collected evidence from existing studies for a network meta-analysis to assess the effectiveness and safety of restarting anticoagulation and different anticoagulation strategies in a population with AF combined with intracranial hemorrhage by direct and indirect comparisons.

## 2. Materials and Methods

This study was conducted in strict adherence to the Preferred Reporting Items for Systematic Reviews and Meta-analysis for Network Meta-analysis (PRISMA-NMA) [12] (Supplementary Methods S1 and S2), and the study protocol was registered with the International Prospective Register of Systematic Reviews (PROSPERO: CRD42024513195).

### 2.1. Data Resources and Search Strategy

We systematically searched PubMed, EMBASE, EBSCO, Cochrane Controlled Trials Central Register (CENTRAL) and ClinicalTrials databases for relevant studies from inception to 1 February 2025 in any language. In addition, we screened the references of the selected literature and the corresponding thematic reviews to further search for any other studies related to the topic and to include those that met the criteria in our study. Details of the search strategy for each database can be found in Supplementary Material S1.

Two authors screened article titles/abstracts independently, and potentially eligible articles were assessed in full text according to inclusion and exclusion criteria. Disagreements were resolved by consensus.

### 2.2. Data Selection Criteria

The inclusion criteria were as follows:

(1) Study design: Randomized controlled trial (RCT) or observational study (case–control or cohort). (2) Participant type: Surviving patients with atrial fibrillation combined with intracranial hemorrhage (without limitation of hemorrhage area and kind of hemorrhage). (3) Intervention: Received any of the following antithrombotic therapies: direct oral anticoagulant (NOAC or OAC), vitamin K antagonist (VKA), antiplatelet, left atrial appendage occlusion (LAAO) or did not receive antithrombotic therapy. (4) Outcome metrics: Thrombosis, recurrent intracranial hemorrhage and all-cause mortality. (5) Where populations overlapped, we included only studies with the largest sample sizes unless different results were provided.

Exclusion criteria: (1) Failure to report outcome metrics of interest to this study. (2) Incompatibility of study type: Literature reviews, case reports, commentaries, conference abstracts, letters and studies for which full text was not available were excluded.

### 2.3. Data Extraction

Two authors independently extracted data on the following aspects according to a standardized form developed, with divergences negotiated with the participation of a third author: (1) Basic information about the study: First author, year of publication, duration of the study, type of study, etc. (2) Patient characteristics: Number of participants, gender, age, country, CHA2DS2-VASc and HAS-BLED scores, etc. (3) Interventions. (4) Outcome indicators and follow-up period.

### 2.4. Quality of Evidence Evaluation and Bias Assessment

The Cochrane tool for assessing the risk of bias in randomized trials (RoB 2) will be used for the quality assessment of randomized studies. In contrast, the risk of bias in non-randomized studies of interventions (ROBINS-I) will be used for the quality assessment of observational studies [13,14]. The risk of bias assessment will be carried out independently by two authors, with disagreements resolved by consensus. We explored possible publication bias by plotting funnel plots and performing Egger’s test [15].

This study assessed the quality of the evidence of the study’s findings using the GRADE rating tool, which evaluates study limitation, indirectness, inconsistency, imprecision and publication. The resulting levels of evidence were “very low”, “low”, “moderate” or “high” [16].

### 2.5. Statistical Analysis

We applied a Bayesian hierarchical model via the Markov chain Monte Carlo (MCMC) algorithm to combine direct and indirect comparisons of treatment strategy outcomes. The convergence of the model was assessed using the Brooks–Gelman–Rubin method, and the model parameters were dynamically adjusted until the convergence factor was close to one [17]. We used a random-effects model for the initial analyses, as random-effects models are likely to be the most appropriate and conservative for explaining within- and between-study variations in the case of direct or indirect comparisons of multiple interventions in multiple studies. The final combined effect values were expressed as odds ratios (ORs) and 95% confidence intervals (CIs). Consistency was tested using the node-split method, which indicates consistency between direct and indirect comparisons when *p* > 0.05. Heterogeneity was tested using the Cochran Q statistic and I^2^. When I^2^ > 50% showed significant heterogeneity, sources of heterogeneity will be sought and discussed through regression and sensitivity analyses [18]. If the combined studies have no considerable heterogeneity, they are adjusted to be analyzed as a fixed-effects model.

We plotted network diagrams and forest plots to visualize direct and indirect comparisons between multiple studies. The surface under the cumulative ranking curve (SUCRA) was used to assess the risk of an outcome occurring, thus evaluating the efficacy and safety of different interventions. The higher the SUCRA score, the better the treatment benefit, and vice versa. The high and low SUCRA scores ranged from 0 (worst) to 1 (best), consistent with the probability of possible ranking corresponding to different interventions [19]. *p*-value < 0.05 was considered statistically different, and all tests were two-tailed.

Statistical analyses and graphing were completed using ADDIS software (Version 1.16.8) and Stata software (Version 18.0).

## 3. Results

**Figure 1:**
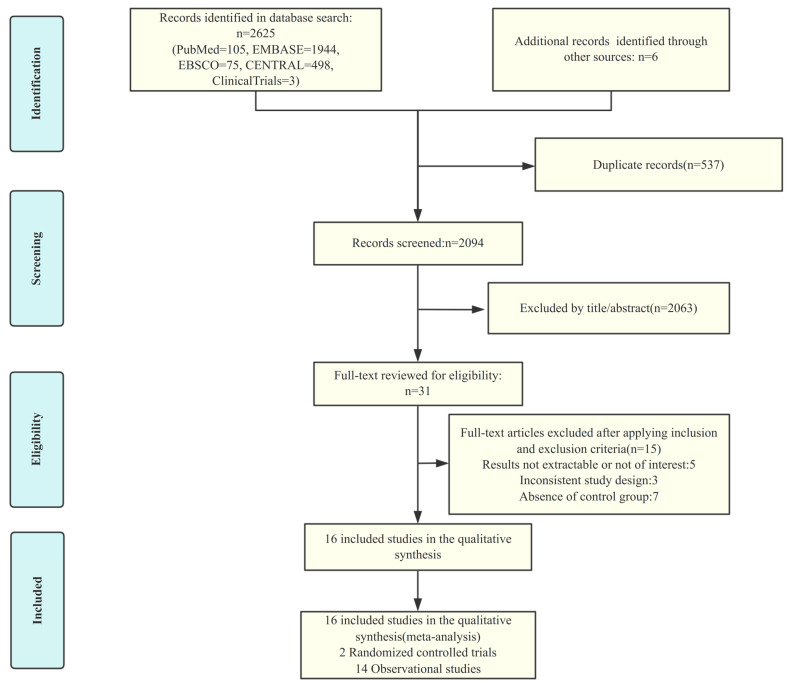
PRISMA flow diagram of study screening and selection.

A total of 2631 papers were retrieved for our study, of which 31 were reviewed in full text. Two randomized controlled trials and 14 observational studies were finally included (Table S6). Figure 1 details the literature screening process and reasons for inclusion or exclusion. A total of 25,483 participants, with a mean age of 75.7 years and a mean percentage of males of 56.47%, were enrolled in the included studies. A total of 2109 thromboembolic events, 1820 recurrent intracranial hemorrhages and 1021 all-cause deaths were observed. Detailed definitions of the endpoint events are provided in Table S7. A total of nine direct comparisons of six different interventions were performed in these studies, including OAC versus no antithrombotic therapy, NOAC versus warfarin, warfarin versus no antithrombotic therapy, antiplatelet versus no antithrombotic therapy, NOAC versus antiplatelet therapy, NOAC versus no antithrombotic therapy, OAC versus antiplatelet therapy, warfarin versus antiplatelet and OAC versus left auricular occlusion (LAAO). Table 1 summarizes the participant characteristics of each study and basic information about the studies.

### 3.1. Risk of Bias Assessment

Bias was assessed for randomized controlled trials and observational studies based on RoB 2 and ROBINS-I, respectively (Supplementary Figure S1). The overall risk of bias for the two randomized controlled trials was moderate, and missing data in the random allocation process was the leading cause of bias. In the risk of bias assessment of observational studies, most studies were at low or medium risk of bias, mainly due to the possibility of confounding bias as they were observational studies. Two studies were at high risk of bias, mainly due to missing data, and the rest of the areas were at low or medium risk of bias. For non-randomized controlled trials, the presence of low to moderate risk of bias is reasonable, so the 14 observational studies we included could provide reasonable evidence.

By assessing publication bias separately for different outcome events, we did not find publication bias for recurrent intracranial hemorrhage (Egger’s test *p* = 0.104), but found publication bias for thromboembolism (Egger’s test *p* = 0.014) and all-cause mortality (Egger’s test *p* = 0.044) (Supplementary Figure S2).

### 3.2. Thromboembolism

**Figure 2:**
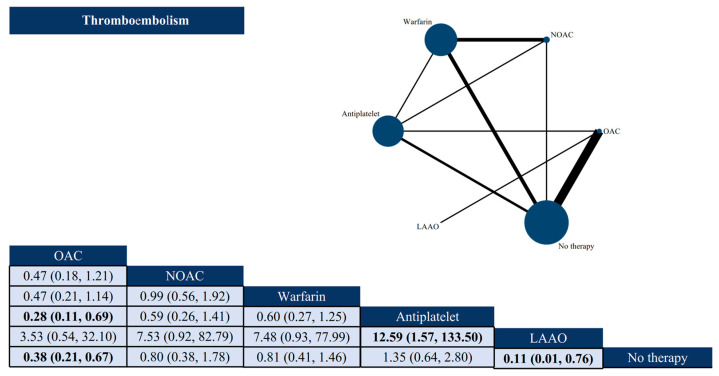
Network plots and league tables of thromboembolism. Network diagram: Nodes represent antithrombotic strategies, and edges represent direct comparisons in included trials. The size of the nodes is proportional to the number of patients per strategy, and the thickness of the edges is proportional to the number of studies with direct comparisons between strategies. League table: Includes antithrombotic strategies and ORs and 95% CIs between column- and row-defining regimens. Bold indicates statistically significant results. OAC: oral anticoagulant; NOAC: new oral anticoagulant; LAAO: left atrial appendage occlusion.

Thromboembolic events were reported in 14 studies with a total of 18,446 patients. Of these, 2109 patients (11.43%) developed thromboembolism (Table S8). Compared with no antithrombotic therapy, OAC (OR: 0.38, 95% CI: 0.21–0.67) and LAAO (OR: 0.11, 95% CI: 0.01–0.76) were associated with a lower risk of thromboembolism. Patients taking OAC (OR: 0.28, 95% CI: 0.11–0.69) had a lower risk of thromboembolism than antiplatelet therapy. Compared with LAAO, antiplatelet therapy (OR: 12.59, 95% CI: 1.57–133.50) had a higher correlation with thromboembolism. There was no statistically significant difference between the two comparisons for the remaining interventions (Figure 2).

### 3.3. Recurrent Intracranial Hemorrhage

**Figure 3:**
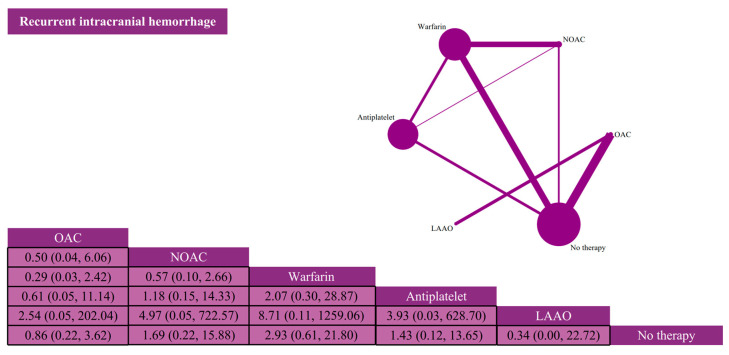
Network plots and league tables of recurrent intracranial hemorrhage. Network plots and league tables of thromboembolism. Network diagram: Nodes represent antithrombotic strategies, and edges represent direct comparisons in included trials. The size of the nodes is proportional to the number of patients per strategy, and the thickness of the edges is proportional to the number of studies with direct comparisons between strategies. League table: Includes antithrombotic strategies and ORs and 95% CIs between column- and row-defining regimens. OAC: oral anticoagulant; NOAC: new oral anticoagulant; LAAO: left atrial appendage occlusion.

Thirteen studies reported recurrent intracranial hemorrhage events involving 18,300 patients. Recurrent intracranial hemorrhage occurred in 1820 of these patients (9.95%) (Table S8). Overall, the probability of recurrent intracranial hemorrhage was similar for the six interventions (Figure 3).

### 3.4. All-Cause Mortality

Eleven studies reported all-cause mortality events involving 4747 patients. Of these, 1021 patients (21.51%) ultimately died (Table S8).

**Figure 4:**
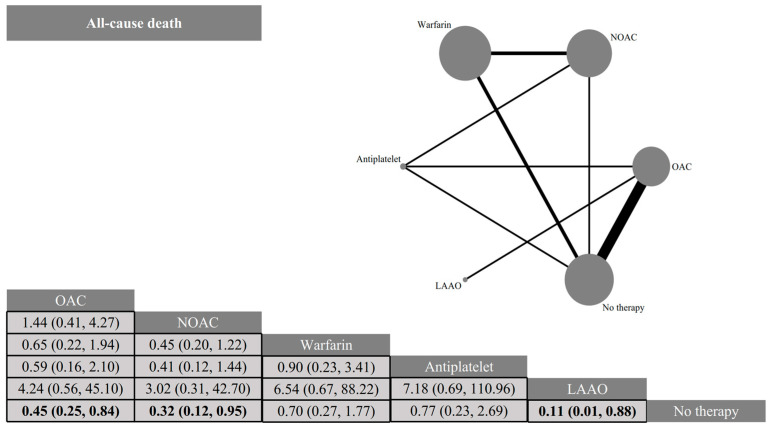
Network plots and league tables of all-cause mortality. Network diagram: Nodes represent antithrombotic strategies, and edges represent direct comparisons in included trials. The size of the nodes is proportional to the number of patients per strategy, and the thickness of the edges is proportional to the number of studies with direct comparisons between strategies. League table: Includes antithrombotic strategies and ORs and 95% CIs between column- and row-defining regimens. Bold indicates statistically significant results. OAC: oral anticoagulant; NOAC: new oral anticoagulant; LAAO: left atrial appendage occlusion.

The risk of all-cause mortality was lower in patients intervening with OAC (OR: 0.45, 95% CI: 0.25–0.84), NOAC (OR: 0.32, 95% CI: 0.12–0.95) and LAAO (OR: 0.11, 95% CI: 0.01–0.88) compared to no antithrombotic therapy. There was no statistically significant difference between the two comparisons for the remaining interventions (Figure 4).

### 3.5. Ranking of Effectiveness and Safety of Antithrombotic Strategies

**Figure 5:**
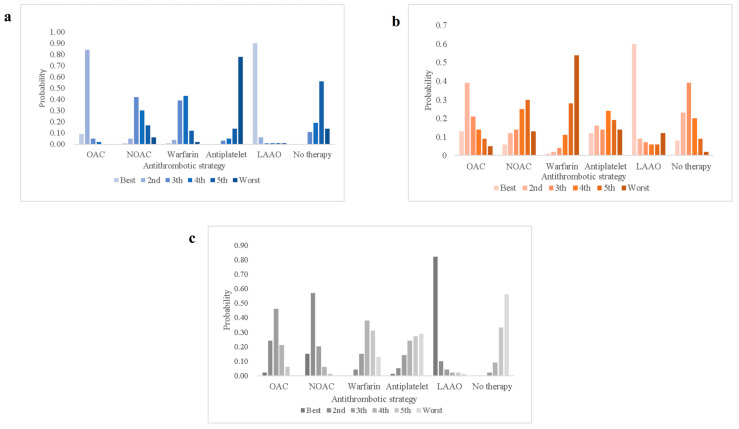
Ranking the likelihood of antithrombotic strategies for different outcomes. Best means the greatest likelihood of preventing the adverse outcome, and Worst means the greatest likelihood of the adverse outcome occurring. OAC: oral anticoagulant; NOAC: new oral anticoagulant; LAAO: left atrial appendage occlusion. (**a**) Thromboembolism; (**b**) recurrent intracranial hemorrhage; (**c**) all-cause mortality.

We ranked the effectiveness and safety of different antithrombotic strategies for patients with AF combined with intracranial hemorrhage. Based on the SUCRA values summarized in Table 2, LAAO consistently shows the highest SUCRA scores for the prevention of thromboembolic events, recurrent intracranial hemorrhage and all-cause mortality, indicating the greatest likelihood of providing optimal therapeutic benefit. We plotted the ranked probability of likelihood that each antithrombotic strategy corresponded to the outcome (Figure 5). Supplementary Figure S3 illustrates the proportion of direct and indirect evidence estimated for each network. Supplementary Figure S4 shows the cumulative probability ranking plots of different intervention strategies for each outcome.

For thromboembolic events, in addition to LAAO (0.96), OAC (0.80) may also provide substantial therapeutic benefit, whereas antiplatelet therapy alone appears to offer poor antithrombotic efficacy (0.06). For recurrent intracranial hemorrhage, LAAO (0.75) is likely the most effective strategy for reducing risk, followed by oral anticoagulation (OAC, 0.64) or no therapy (0.60). However, warfarin (0.15) had the highest likelihood of bleeding risk and the lowest safety benefit. For all-cause mortality, LAAO (0.94) was the preferred strategy to reduce the incidence of death, followed by NOAC (0.75). The likelihood of death due to no antithrombotic intervention (0.11) was the highest.

### 3.6. Consistency and Heterogeneity Tests for Network

#### 3.6.1. Consistency

All of our fitted models converged well, and consistency tests were performed by node-splitting, with all *p*-value > 0.05, and no evidence of inconsistency was found between direct and indirect comparisons (Table S9).

#### 3.6.2. Heterogeneity Test

We performed separate tests for heterogeneity to pool studies with different outcome events. We found varying degrees of heterogeneity for all three outcome events combined. There was significant heterogeneity in the subgroups of the two-by-two comparisons of the OAC versus no antithrombotic therapy group and the warfarin versus no antithrombotic therapy group. Regression analyses did not identify the duration of follow-up, sex or propensity score as sources of heterogeneity. Across the included studies, there was no standardized reporting of anticoagulation resumption timing after ICH, despite its critical role in determining both thromboembolic risk and the likelihood of recurrent hemorrhage. This inconsistency limited our ability to systematically assess its impact within the regression models. In addition, the anatomical location of ICH (lobar vs. deep) differs in underlying pathology and recurrence risk, which may influence clinical decision-making and contribute to variability across studies. Variations in AF subtype (valvular vs. non-valvular) may also affect baseline thromboembolic risk and anticoagulation strategy selection, thereby introducing additional heterogeneity. Moreover, the LAAO-related studies included in our analysis were observational in nature, which may increase the potential for confounding inherent to non-randomized designs and further contribute to heterogeneity. Our analyses indicated that study type and study design were the primary contributors to heterogeneity. Nevertheless, sensitivity analyses performed after excluding the relevant studies demonstrated that the overall results remained stable, supporting the robustness of our findings. (Table S10, Supplementary Figures S5 and S6).

## 4. Discussion

This study conducted a Bayesian NMA on the resumption of anticoagulation and the choice of anticoagulation strategy in patients with AF after intracranial hemorrhage. To the best of our knowledge, this study is the first to compare the efficacy and safety of different anticoagulant treatments, antiplatelet therapy, left auricular occlusion and the absence of any antithrombotic interventions in patients with AF combined with intracranial hemorrhage. The GRADE level of evidence was rated as moderate quality (Table S11). Our NMA results from 16 studies of 25,483 participants showed that OAC and LAAO were associated with a lower risk of thromboembolism and all-cause mortality and did not significantly increase the risk of intracranial hemorrhage compared with no antithrombotic intervention. However, antiplatelet therapy was associated with an increased risk of thromboembolism compared with OAC and LAAO therapy and no advantage in reducing the risk of intracranial hemorrhage findings. In addition, SUCRA and league table showed that LAAO may be the best strategy for preventing thromboembolism and reducing the recurrence of intracranial hemorrhage and all-cause mortality in patients surviving AF with intracranial hemorrhage. At the same time, recurrence of intracranial hemorrhage due to warfarin therapy is most likely, and the absence of any antithrombotic treatment is highly likely to lead to death from any cause in patients.

Achieving the optimal balance between preventing thromboembolism and preventing rebleeding (especially recurrent intracranial hemorrhage) in patients with AF after intracranial hemorrhage is a challenging task for clinicians. Many AF patients with a history of previous intracranial hemorrhage refuse OAC for fear of recurrence of intracranial hemorrhage [35,36], creating a clinical dilemma of stroke and embolism prevention in these patients, and LAAO may be the solution to this dilemma. LAAO is increasingly being used to reduce the risk of stroke and hemorrhage in patients with AF and is superior to OAC for the combined endpoint of stroke, hemorrhage and all-cause mortality [37,38,39]. Despite this, head-to-head comparisons of LAAO with other antithrombotic interventions in patients with AF combined with intracranial hemorrhage are scarce. Our study included a study comparing the clinical outcomes of LAAO and standard pharmacological therapy in patients with AF with prior ICH, suggesting a potential therapeutic benefit of LAAO in such patients [34]. In direct and indirect comparisons in this study, LAAO was the primary choice regarding the effectiveness and safety of antithrombotic strategies with lower risk of thromboembolism, recurrent intracranial hemorrhage and all-cause mortality.

The results of several previous observational studies have shown that anticoagulation reduces the risk of ischemic stroke or vascular embolism and all-cause mortality [27,28,32]. Previous meta-analyses have reported support for the effectiveness of OAC (including NOAC and warfarin) in reducing thromboembolic events and all-cause mortality [40,41]. Based on the available data integrated, our study extends this finding, thus observing the effectiveness of anticoagulants in patients after intracranial hemorrhage. Our study suggests no significant difference in the incidence of recurrent intracranial hemorrhage in patients in each intervention group. Still, in contrast, the likelihood of recurrent intracranial hemorrhage due to warfarin was the highest, in line with the conclusions of several meta-analyses and RCTs [21,22,41,42]. These results suggest that anticoagulant medication may not be a catalyst for secondary intracranial hemorrhage while affirming the value of OAC, particularly NOAC, in such patients.

Observational studies have pointed out that the use of antiplatelet agents for AF patients with a previous history of ICH does not significantly reduce the risk of ischemic stroke but increases or does not reduce the risk of developing ICH [26,32]. Our study, comparing multiple antithrombotic strategies directly or indirectly with antiplatelet therapy, found that antiplatelet therapy may increase the risk of thromboembolism but did not reduce the risk of bleeding in patients. Therefore, antiplatelet therapy cannot be used as a primary antithrombotic option for this particular group of patients with AF.

It is important to note that our study found a similar risk of recurrent intracranial hemorrhage for all head-to-head interventions compared. The potential reasons for this may be multifaceted; on the one hand, the short follow-up period, with the vast majority of included studies having a follow-up period of ≤1 year and no more than 3 years, may not have exposed significant differences in intracranial hemorrhage across intervention strategies. Secondly, the definition of recurrence of intracranial hemorrhage and inclusion criteria varied slightly between studies. In addition, the clinician’s assessment of the patient’s risk of rebleeding may influence the prescription of interventions.

The clinical issue of resuming anticoagulation in patients with atrial fibrillation after intracranial hemorrhage is not clearly defined and is mainly based on the clinician’s judgment of the patient’s risk of thromboembolism and the likelihood of recurrent hemorrhage. Our study compares the efficacy and safety of whether or not to resume anticoagulation in patients with AF combined with intracranial hemorrhage and proposes that the resumption of anticoagulation is the first decision in this group of patients, providing evidence for clinical decision-making. Second, this study is the first comprehensive pooled analysis comparing the effectiveness and safety of various antithrombotic strategies. Several systematic reviews and meta-analyses based on randomized controlled trials have comprehensively evaluated the long-term efficacy and safety of LAAO, OACs and VKAs in patients with atrial fibrillation. These studies consistently demonstrate that, compared with OACs and VKAs, LAAO is associated with significant reductions in all-cause mortality, hemorrhagic stroke and non-procedural bleeding during long-term follow-up, without an observed increase in thromboembolic events [43,44]. These findings provide important evidence supporting LAAO as a viable antithrombotic strategy. Distinct from prior analyses that primarily evaluated outcomes such as ‘hemorrhagic stroke,’ our study specifically focuses on a high-risk and relatively understudied subgroup—AF patients with a history of hemorrhagic stroke. By systematically comparing multiple antithrombotic strategies, including LAAO and OACs (both DOACs and VKAs), within this vulnerable population, our results extend and complement the existing body of evidence. We further highlight the potential advantages of LAAO over other currently available stroke-prevention strategies in this special cohort. In addition, building upon previous meta-analyses, our study incorporates both direct and indirect comparisons, thereby providing a more comprehensive assessment of the effectiveness and safety of OACs.

Our study also has some limitations. First, most of the included studies were observational, with potential confounding biases, including patients’ age, comorbidities, caste and autonomy intentions. These factors may influence physicians’ intervention decisions in the real world. Second, there are fewer comparisons of LAAO with other interventions, and some studies are still in progress (Supplementary Material S3). Although the inclusion of clinical outcome data from the LAAO intervention in this study suggests a therapeutic advantage of LAAO, the results obtained have to be interpreted with caution, and more RCTs are needed to validate them in the future. In addition, important clinical uncertainties remain regarding the peri-procedural risks and long-term prognostic implications of LAAO, including peri-device leaks (PDLs), device-related thrombus and inconsistencies in the criteria used to evaluate procedural success [45]. These factors may influence the true net clinical benefit of LAAO in AF patients with a history of ICH. Therefore, future studies should more systematically assess peri-procedural safety and long-term outcome measures associated with LAAO to ensure more precise and evidence-based application of this strategy in this high-risk population. Third, because it was a pooled analysis of multiple studies, the individual outcome definitions varied slightly, including thromboembolism (some studies provided data on cerebral ischemic stroke and some on cerebral ischemic stroke and peripheral vascular embolism) and recurrence of intracranial hemorrhage (with varying location and hematoma size). The incidence of outcome events obtained from different studies with different follow-up times varied, which may have affected the pooled analyses. Fourth, some studies did not differentiate between NOAC and warfarin, so while we pooled studies comparing both OAC and NOAC in our study, more studies are needed to directly compare the efficacy and safety of NOAC and warfarin and refine the therapeutic benefits of OAC. Fifth, due to the limited number of available studies, our analysis could not incorporate more granular information regarding anticoagulant dosing, the optimal timing for anticoagulation resumption after ICH or the comparative effectiveness and safety of adjunctive antithrombotic regimens following LAAO. In addition, the evidence base for this meta-analysis is derived primarily from traditional randomized controlled trials and observational studies. With the rapid advancement of machine learning-based risk prediction tools and individualized treatment models, these emerging approaches are increasingly influencing clinical decision-making in post-ICH antithrombotic management. The absence of studies employing such technologies restricts the breadth of evidence included in our analysis. Future research should integrate AI-driven risk stratification methods and personalized therapeutic strategies and, on this basis, conduct higher-quality randomized controlled trials to further optimize antithrombotic management for this high-risk population.

## 5. Conclusions

In this NMA, the resumption of anticoagulation and multiple anticoagulation strategies were compared in patients with AF after intracranial hemorrhage. Resumption of anticoagulation is the best option for such patients to reduce the risk of all types of adverse events and death. LAAO may be the best strategy among all types of stroke prevention interventions for AF. Compared with no intervention and antiplatelet therapy, OAC and LAAO were effective in reducing the risk of thromboembolism and all-cause mortality, and there was no difference in the risk of recurrence of intracranial hemorrhage. In contrast, warfarin in OAC has the potential to increase the risk of recurrence of intracranial hemorrhage, whereas NOAC may be superior in preventing recurrence of intracranial hemorrhage. Overall, these findings may provide therapeutic guidance for AF survivors with intracranial hemorrhage. It is important to note that the evidence from these studies is primarily from observational studies and reflects more real-world data. Therefore, more direct comparative validation is needed, particularly on the effectiveness and safety of LAAO compared with other interventions in this patient population.

## Figures and Tables

**Table 1:** Characteristics of included studies.

First Author, Year	Study Design	Sample Size (n)	Average Age	Male (%)	Mean CHA2DS2-VASc	Mean HAS-BLED	Intervention	Average Follow-Up	Propensity
Satoshi Suda, 2023 [20]	Retrospective cohort	160	77.0	67.50%	2.5	3	OAC vs. No therapy	within 3 months	No
Wu, Victor Chien-Chia, 2021 [11]	Retrospective cohort	372	71.3	47.85%	4.2	2.5	OAC vs. No therapy	1 year	Yes
Floris H B M Schreuder, 2021 [21]	RCT	101	78.0	54.00%	4	NA	NOAC vs. Antiplatelet	1.9 years	Yes
Rustam Al-Shahi Salman, 2021 [22]	RCT	203	79.0	63.00%	4	2	OAC vs. No therapy	1.2 years	Yes
Alireza Sadighi, 2020 [23]	Prospective cohort	93	76.2	54.30%	NA	NA	OAC vs. No therapy	25.8 months	No
Peter Brønnum Nielsen, 2019 [24]	Retrospective cohort	622	76.0	61.00%	4.4	NA	NOAC vs. Warfarin	3 years	Yes
Mohammed K. Badi, 2019 [25]	Retrospective cohort	308	76.0	64.90%	5.7	4.7	NOAC vs. Warfarin vs. No therapy	9.93 months	No
Loris Poli, 2018 [26]	Prospective cohort	146	77.6	60.27%	NA	NA	OAC vs. Antiplatelet vs. No therapy	18 months	Yes
Peter Brønnum Nielsen, 2017 [27]	Prospective cohort	1325	76.8	56.70%	4.1	3.7	Warfarin vs. No therapy	1 year	Yes
Gye Young Park, 2016 [28]	Retrospective cohort	428	68.5	34.11%	3.3	3.5	Warfarin vs. No therapy	34.2 months	No
Chuan-Tsai Tsai, 2020 [29]	Retrospective cohort	1946	75.6	53.70%	5.5	4.3	NOAC vs. Warfarin	within 3 months	No
So-Ryoung Lee, 2020 [30]	Retrospective cohort	5704	73.5	57.20%	4	4.4	NOAC vs. Warfarin	0.6 years	Yes
Sylvie Perreault, 2019 [31]	Retrospective cohort	683	83.0	53.15%	3.9	2.6	OAC vs. No therapy	1 year	No
Tze-Fan Chao, 2016 [32]	Retrospective cohort	12,917	74.7	42.73%	6	NA	Warfarin vs. Antiplatelet vs. No therapy	within 3 months	No
Kuramatsu J.B., 2015 [33]	Retrospective cohort	261	73.7	71.00%	2	NA	OAC vs. No therapy	1 year	Yes
Jens Erik Nielsen-Kudsk 2017 [34]	Retrospective cohort	206	73.9	62.10%	3.9	4.2	OAC vs. LAAO	within 3 months	Yes

Abbreviations: RCT: randomized controlled trials, OAC: oral anticoagulation, NOAC: new oral anticoagulation, LAAO: left atrial appendage occlusion, NA: not available.

**Table 2:** SUCRA scores for each antithrombotic strategy and endpoint.

Antithrombotic Strategy	Thromboembolism	Recurrent Intracranial Hemorrhage	All-Cause Death
OAC	0.80	0.64	0.58
NOAC	0.46	0.40	0.75
Warfarin	0.47	0.15	0.34
Antiplatelet	0.06	0.47	0.28
LAAO	**0.96**	**0.75**	**0.94**
No therapy	0.25	0.60	0.11

Abbreviations: SUCRA: surface under the cumulative ranking curve; OAC: oral anticoagulation; NOAC: new oral anticoagulation; LAAO: left atrial appendage occlusion. The bolded data in the table represents the highest scores.

## Data Availability

All data used in this study are from original, publicly available research, and corresponding references can be found in the Supplementary Materials.

## Supplementary Materials

The following supporting information can be downloaded at https://www.mdpi.com/article/10.3390/jcdd12120464/s1, More detailed data for this study can be obtained from Supplementary Materials S1–S3. S1: Table S1: PubMed database search strategies; Table S2: EMBASE database search strategies; Table S3: EBSCO database search strategies; Table S4: Cochrane Central Register of Controlled Trials (CENTRAL) database search strategies; Table S5: Clinical Trials database search strategies. S2: Table S6: Included studies; Table S7: Definitions of thromboembolism and recurrent intracranial hemorrhage; Table S8: Direct comparison of the number of outcome events; Table S9: Consistency tests for direct and indirect comparisons; Table S10: Meta-regression analysis of different factors for each outcome; Table S11: GRADE assessment of the quality of evidence from network meta-analysis results; Figure S1: Risk of bias assessment of included studies; Figure S2: Publication bias assessment; Figure S3: Direct and indirect evidence proportion for each network estimate; Figure S4: Cumulative probability ranking plots of different intervention strategies for each outcome; Figure S5: Direct comparison of forest plots for each outcome; Figure S6: Sensitivity analysis; Supplementary Methods S1: PRISMA Main Checklist; Supplementary Methods S2: PRISMA Abstract Checklist. Supplementary Methods S3: ClinicalTrials.gov Search Results 1 February 2025.
