# Supplementary material for: Efficacy and Safety of Drug and Device Strategies for Stroke Prevention in Atrial Fibrillation After Intracranial Hemorrhage: A Bayesian Network Meta-Analysis
Source: J Cardiovasc Dev Dis. 2025 Nov 28;12(12):464. doi: 10.3390/jcdd12120464 (PMC12733742; doi:10.3390/jcdd12120464)
Supplement: Supplementary file 1 [file jcdd-12-00464-s001.zip › Supplementary material S2.pdf]

Supplemental material

Table S6 Included studies
Table S7 Definitions of thromboembolism and recurrent intracranial hemorrhage
Table S8 Direct comparison of the number of outcome events
Table S9 Consistency tests for direct and indirect comparisons
Table S10 Meta-regression analysis of different factors for each outcome
Table S11 GRADE assessment of the quality of evidence from network meta-analysis results
Supplementary Figure S1 Risk of bias assessment of included studies
Supplementary Figure S2 Publication bias assessment
Supplementary Figure S3 Direct and indirect evidence proportion for each network estimate
Supplementary Figure S4 Cumulative probability ranking plots of different intervention strategies for each outcome
Supplementary Figure S5 Direct comparison of forest plots for each outcome
Supplementary Figure S6 Sensitivity analysis
Supplementary Methods S1. PRISMA Main Checklist
Supplementary Methods S2. PRISMA Abstract Checklist
References

Table S6 Included studies

The network meta-analysis included 2 randomized controlled trials and 14 observational studies.

First Author,Year[Reference]	Study design
Floris H B M Schreuder,2021[1]	RCT
Rustam Al-Shahi Salman,2021[2]	RCT
Satoshi Suda,2023[3]	Retrospective cohort
Wu, Victor Chien-Chia,2021[4]	Retrospective cohort
Alireza Sadighi,2020[5]	Prospective cohort
Peter Brønnum Nielsen,2019[6]	Retrospective cohort
Mohammed K. Badi,2019[7]	Retrospective cohort
Loris Poli,2018[8]	Prospective cohort
Peter Brønnum Nielsen,2017[9]	Prospective cohort
Gye Young Park,2016[10]	Retrospective cohort
Chuan-Tsai Tsai,2020[11]	Retrospective cohort
So-Ryoung Lee,2020[12]	Retrospective cohort
Sylvie Perreault,2019[13]	Retrospective cohort
Tze-Fan Chao,2016[14]	Retrospective cohort
Kuramatsu JB,2015[15]	Retrospective cohort
Jens Erik Nielsen-Kudsk1,2017[16]	Retrospective cohort

Table S7 Definitions of thromboembolism and recurrent intracranial hemorrhage

Study	Thromboembolism	Recurrent intracranial hemorrhage
Satoshi Suda,2023	Ischemic stroke,deep venous thrombosis	Imaging aids in the diagnosis of new intracranial haemorrhage
Wu, Victor Chien-Chia,2021	Ischemic stroke & systemic embolism	It was defined according to the principal diagnosis at admission or an emergency visit during which IS was previously validated
Floris H B M Schreuder,2021	Ischemic stroke	Clinical evidence of the sudden onset of a new neurological deficit, or an increase in an existing deficit, persisting for more than 24 hours,with a corresponding intracerebral haemorrhage on a CT or MR scan or at post-mortem investigation
Rustam Al-Shahi Salman,2021	Ischemic stroke	Recurrent symptomatic spontaneous intracranial haemorrhage
Alireza Sadighi,2020	Ischemic stroke & systemic embolism	Definition missing
Peter Brønnum Nielsen,2019	Ischemic stroke	The 10th revision of ICD codes was used to identify outcomes:ICD: I61
Mohammed K. Badi,2019	Ischemic stroke	It wasdefined as nontraumatic intracerebral hemorrhage identified on brain imaging)
Loris Poli,2018	ischemic stroke/systemic embolism	NA
Peter Brønnum Nielsen,2017	Ischemic stroke & systemic embolism	Definition missing
Gye Young Park,2016	Ischemic strokes, pulmonary thromboembolism, deep vein thrombosis, or other systemic embolisms	Recurrent CNS bleeding
Chuan-Tsai Tsai,2020	Ischemic stroke	Definition missing
So-Ryoung Lee,2020	Ischemic stroke	NA
Sylvie Perreault,2019	Ischemic stroke,fatal ischemic stroke	intracranial hemorrhage,fatal intracranial hemorrhage
Tze-Fan Chao,2016	Ischemic stroke	Definition missing
Kuramatsu JB,2015	New cerebral Infarction	Definition missing
Jens Erik Nielsen-Kudsk1,2017	Ischemic stroke	Definition missing

Table S8 Direct comparison of the number of outcome events

Thromboembolism

Study	Intervention	Events	Total
Satoshi Suda,2023	OAC	5	108
Satoshi Suda,2023	No therapy	2	52
Wu, Victor Chien-Chia,2021	OAC	5	186
Wu, Victor Chien-Chia,2021	No therapy	15	186
Floris H B M Schreuder,2021	NOAC	6	50
Floris H B M Schreuder,2021	Antiplatelet	6	51
Rustam Al-Shahi Salman,2021	OAC	3	101
Rustam Al-Shahi Salman,2021	No therapy	21	102
Alireza Sadighi,2020	OAC	7	38
Alireza Sadighi,2020	No therapy	10	55
Peter Brønnum Nielsen,2019	NOAC	29	348
Peter Brønnum Nielsen,2019	Warfarin	32	274
Mohammed K. Badi,2019	NOAC	16	88
Mohammed K. Badi,2019	Warfarin	18	128
Mohammed K. Badi,2019	No therapy	17	92
Loris Poli,2018	OAC	2	55
Loris Poli,2018	Antiplatelet	8	29
Loris Poli,2018	No therapy	13	62
Gye Young Park,2016	Warfarin	9	254
Gye Young Park,2016	No therapy	17	174
Chuan-Tsai Tsai,2020	NOAC	72	973
Chuan-Tsai Tsai,2020	Warfarin	76	973
Sylvie Perreault,2019	OAC	6	260
Sylvie Perreault,2019	No therapy	11	423
Tze-Fan Chao,2016	Warfarin	130	1154
Tze-Fan Chao,2016	Antiplatelet	581	3552
Tze-Fan Chao,2016	No therapy	964	8211
Kuramatsu JB,2015	OAC	4	108
Kuramatsu JB,2015	No therapy	16	153
Jens Erik Nielsen-Kudsk1,2017	OAC	6	103
Jens Erik Nielsen-Kudsk1,2017	LAAO	2	103

Recurrent intracranial hemorrhage

Study	Intervention	Events	Total
Satoshi Suda,2023	OAC	0	108
Satoshi Suda,2023	No therapy	0	52
Wu, Victor Chien-Chia,2021	OAC	3	186
Wu, Victor Chien-Chia,2021	No therapy	7	186
Floris H B M Schreuder,2021	NOAC	4	50
Floris H B M Schreuder,2021	Antiplatelet	1	51
Rustam Al-Shahi Salman,2021	OAC	8	101
Rustam Al-Shahi Salman,2021	No therapy	4	102
Alireza Sadighi,2020	OAC	5	38
Alireza Sadighi,2020	No therapy	3	55
Peter Brønnum Nielsen,2019	NOAC	27	348
Peter Brønnum Nielsen,2019	Warfarin	22	274
Mohammed K. Badi,2019	NOAC	1	88
Mohammed K. Badi,2019	Warfarin	6	128
Mohammed K. Badi,2019	No therapy	6	92
Gye Young Park,2016	Warfarin	13	254
Gye Young Park,2016	No therapy	0	174
Chuan-Tsai Tsai,2020	NOAC	26	973
Chuan-Tsai Tsai,2020	Warfarin	46	973
Sylvie Perreault,2019	OAC	4	260
Sylvie Perreault,2019	No therapy	23	423
Tze-Fan Chao,2016	Warfarin	241	1154
Tze-Fan Chao,2016	Antiplatelet	628	3552
Tze-Fan Chao,2016	No therapy	730	8211
Kuramatsu JB,2015	OAC	4	108
Kuramatsu JB,2015	No therapy	5	153
Jens Erik Nielsen-Kudsk1,2017	OAC	2	103
Jens Erik Nielsen-Kudsk1,2017	LAAO	1	103

All-cause death

Study	Intervention	Events	Total
Wu, Victor Chien-Chia,2021	OAC	21	186
Wu, Victor Chien-Chia,2021	No therapy	37	186
Floris H B M Schreuder,2021	NOAC	9	50
Floris H B M Schreuder,2021	Antiplatelet	11	51
Rustam Al-Shahi Salman,2021	OAC	15	101
Rustam Al-Shahi Salman,2021	No therapy	11	102
Alireza Sadighi,2020	OAC	10	38
Alireza Sadighi,2020	No therapy	20	55
Mohammed K. Badi,2019	NOAC	14	88
Mohammed K. Badi,2019	Warfarin	40	128
Mohammed K. Badi,2019	No therapy	33	92
Loris Poli,2018	OAC	5	55
Loris Poli,2018	Antiplatelet	7	29
Loris Poli,2018	No therapy	12	62
Gye Young Park,2016	Warfarin	13	254
Gye Young Park,2016	No therapy	22	174
Chuan-Tsai Tsai,2020	NOAC	187	973
Chuan-Tsai Tsai,2020	Warfarin	389	973
Sylvie Perreault,2019	OAC	20	260
Sylvie Perreault,2019	No therapy	80	423
Kuramatsu JB,2015	OAC	9	108
Kuramatsu JB,2015	No therapy	47	153
Jens Erik Nielsen-Kudsk1,2017	OAC	7	103
Jens Erik Nielsen-Kudsk1,2017	LAAO	2	103

Table S9 Consistency tests for direct and indirect comparisons

Thromboembolism

P>0.05

Intervention	P-Value
Antiplatelet , NOAC	0.41
Antiplatelet , No therapy	0.88
Antiplatelet , OAC	0.14
Antiplatelet , Warfarin	0.64
NOAC, No therapy	0.83
NOAC, Warfarin	0.46
No therapy, Warfarin	0.26

Recurrent intracranial hemorrhage

P>0.05

Intervention	P-Value
Antiplatelet , NOAC	0.23
Antiplatelet , No therapy	0.69
Antiplatelet , Warfarin	0.59
NOAC, No therapy	0.06
NOAC, Warfarin	0.46

All-cause death

P>0.05

Intervention	P-Value
Antiplatelet , NOAC	0.19
Antiplatelet , No therapy	0.65
Antiplatelet , OAC	0.55
NOAC, No therapy	0.84
NOAC, Warfarin	0.21
No therapy , Warfarin	0.4

Table S10 Meta-regression analysis of different factors for each outcome

Factors	Outcome	Effect size	p-value
	Thrombosis		
Follow-up		-0.8	0.439
Prosperity		1	0.331
Sex		-0.65	0.524
	Recurrent intracranial hemorrhage		
Follow-up		1.35	0.203
Prosperity		-0.07	0.942
Sex		1.1	0.293
	All-cause death		
Follow-up		0.78	0.458
Prosperity		-0.42	0.686
Sex		-0.94	0.37

Table S11 GRADE assessment of the quality of evidence from network meta-analysis results

Comparison	Nature of the evidence	Confidence	Reason for downgrading	Reason for upgrading
OAC vs NOAC	Indirect	Low		
OAC vs Warfarin	Indirect	Low		
OAC vs Antiplatelet	Mixed	Low		
OAC vs LAAO	Mixed	Moderate	Imprecision	
OAC vs No therapy	Mixed	Moderate	Study limitations	
NOAC vs Warfarin	Mixed	Low		
NOAC vs Antiplatelet	Mixed	Moderate	Imprecision	
NOAC vs LAAO	Indirect	Moderate		Large magnitude of effect
NOAC vs No therapy	Mixed	Moderate	Imprecision	
Warfarin vs Antiplatelet	Mixed	Moderate	imprecision	
Warfarin vs LAAO	Indirect	Moderate		Large magnitude of effect
Warfarin vs No therapy	Mixed	Moderate	Study limitations	
Antiplatelet vs LAAO	Indirect	Moderate		Large magnitude of effect
Antiplatelet vs No therapy	Mixed	Low		
LAAO vs No therapy	Indirect	Low		
**Total ranking of treatments**		Low	Study limitations	

Supplementary Figure S1 Risk of bias assessment of included studies

Supplementary Figure S1A. Risk of Bias in Non-randomized Studies of Interventions (ROBINS-I)

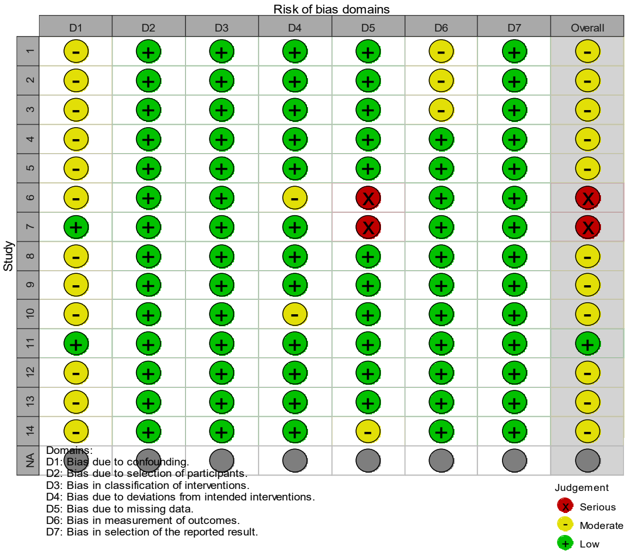

Study1:Satoshi Suda,2023,**2**:Wu,Victor Chien-Chia,2021,**3**:Alireza Sadighi,2020,**4**:Peter Brønnum Nielsen,2019,**5**: Mohammed K. Badi,2019,**6**: Loris Poli,2018,**7**: Peter Brønnum Nielsen,2017,**8**: Gye Young Park,2016,**9**: Chuan-Tsai Tsai,2020,**10**: So-Ryoung Lee,2020,**11**: Sylvie Perreault,2019,**12**: Tze-Fan Chao,2016,**13**: Kuramatsu JB,2015,**14**: Jens Erik Nielsen-Kudsk1,2017.

Supplementary Figure S1B. RoB 2 Cochrane tool for assessing the risk of bias in randomized clinical trials

	D1	D2	D3	D4	D5	Overall
Floris H B M Schreuder.2021	+	!	+	+	!	!
Rustam Al-Shahi Salman.2021	+	+	-	+	!	-

Domains	D1	Randomisation process	Judgement	
	D2	Deviations from the intended interventions	+	Low risk
	D3	Missing outcome data	!	Some concerns
	D4	Measurement of the outcome	-	High risk
	D5	Selection of the reported result		

Supplementary Figure S2. Publication Bias Assessment

Supplementary Figure S2A Thromboembolism

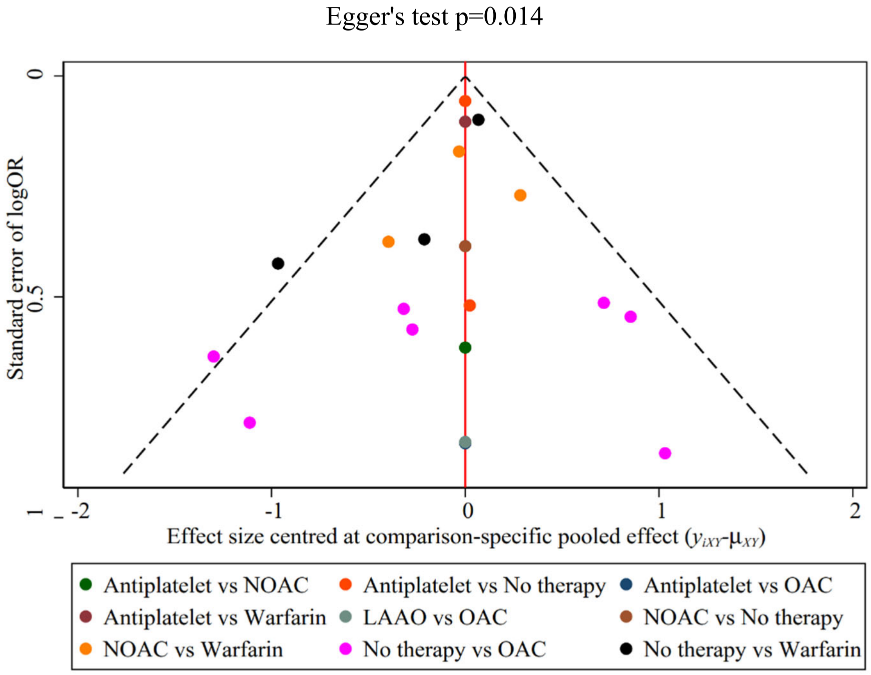

Supplementary Figure S2B Recurrent intracranial hemorrhage

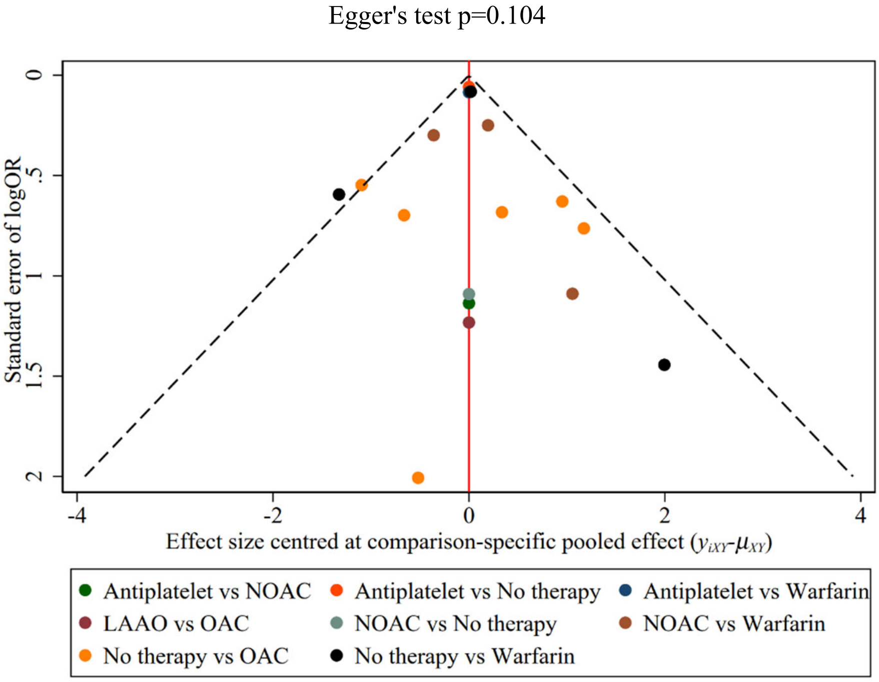

Supplementary Figure S2C All-cause death

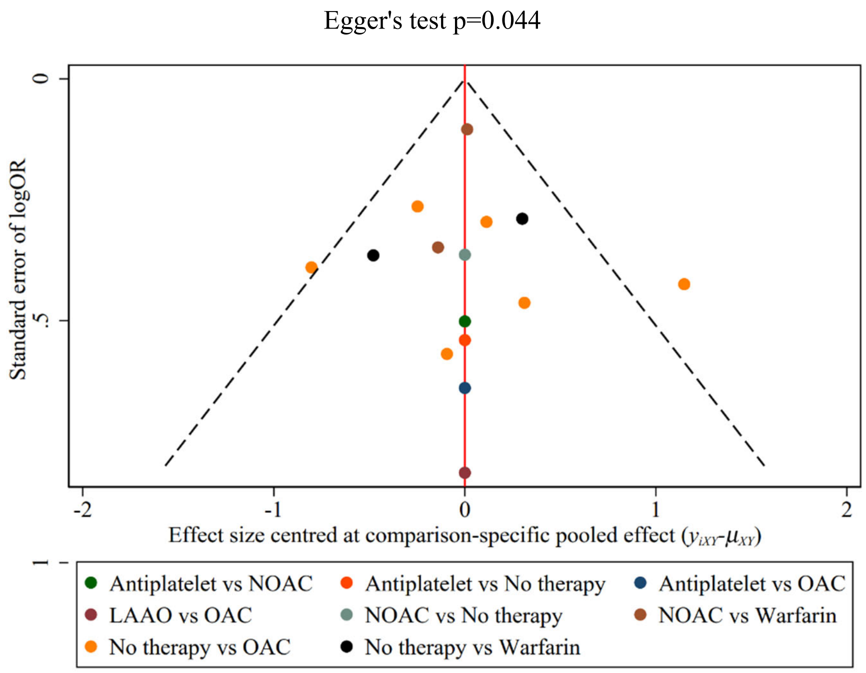

The horizontal axis displays the study's effect quantity. The standard error of the logOR is represented on the vertical. The red line indicates the amount of combined effect. The oblique dotted lines on both sides represent 95% confidence intervals.

Supplementary Figure S3 Direct and indirect evidence proportion for each network estimate

Supplementary Figure S3A Thromboembolism

Direct comparisons in the network

Network meta-analysis estimates	1vs4	1vs5	1vs6	2vs3	2vs4	2vs6	3vs4	3vs6	4vs6
Mixed estimates									
1vs4	7.6	·	44.9	0.6	0.1	0.7	2.0	1.4	42.8
1vs5	·	99.9	·	·	·	·	·	·	·
1vs6	12.2	·	74.8	0.2	·	0.2	0.5	0.4	11.7
2vs3	·	·	·	67.2	3.6	9.1	11.0	1.7	7.4
2vs4	·	·	·	38.4	3.2	8.0	34.5	3.9	11.8
2vs6	0.1	·	0.1	30.7	2.5	7.1	26.3	4.4	28.7
3vs4	·	·	·	5.5	1.6	3.9	68.7	8.2	12.0
3vs6	0.2	·	0.2	4.2	0.9	3.3	41.8	6.9	42.6
4vs6	0.4	·	0.4	1.3	0.2	1.4	4.1	2.8	89.5
Indirect estimates									
1vs2	4.3	·	25.6	22.8	1.9	5.2	19.7	3.1	17.3
1vs3	5.2	·	31.1	2.8	0.7	2.2	29.0	4.6	24.4
2vs5	3.3	23.0	19.7	17.6	1.5	4.0	15.2	2.4	13.3
3vs5	3.8	26.7	22.8	2.1	0.5	1.6	21.2	3.3	17.9
4vs5	5.0	34.4	29.4	0.4	0.1	0.5	1.3	0.9	28.1
5vs6	6.5	46.5	40.0	0.1	·	0.1	0.3	0.2	6.3
**Entire network**	3.5	15.1	20.7	12.8	1.1	3.2	18.5	3.0	22.1
**Included studies**	1	1	7	3	1	1	1	3	2

Supplementary Figure S3B Recurrent intracranial hemorrhage

Direct comparisons in the network

Network meta-analysis estimates	1vs5	1vs6	2vs3	2vs4	2vs6	3vs4	3vs6	4vs6
Mixed estimates								
1vs5	100.0	·	·	·	·	·	·	·
1vs6	0.1	99.8	·	·	·	0.1	·	0.1
2vs3	·	·	78.7	4.1	4.4	8.3	0.2	4.2
2vs4	·	·	45.4	2.6	2.9	44.6	0.8	3.6
2vs6	·	·	31.7	1.8	2.1	30.8	0.9	32.7
3vs4	·	·	1.0	0.5	0.5	94.1	1.7	2.2
3vs6	·	·	0.6	0.2	0.4	48.6	1.3	48.8
4vs6	·	·	0.2	·	0.3	1.1	0.8	97.6
Indirect estimates								
1vs2	·	26.3	23.4	1.4	1.5	22.7	0.6	24.1
1vs3	·	33.6	0.4	0.2	0.3	32.3	0.9	32.4
1vs4	·	49.6	0.1	·	0.1	0.6	0.4	49.1
2vs5	20.8	20.8	18.5	1.1	1.2	18.0	0.5	19.1
3vs5	25.1	25.1	0.3	0.1	0.2	24.2	0.7	24.3
4vs5	33.2	33.2	0.1	·	0.1	0.4	0.3	32.8
6vs5	50.0	49.9	·	·	·	·	·	·
**Entire network**	14.5	23.1	13.1	0.8	0.9	21.6	0.6	25.4
**Included studies**	1	6	3	1	1	1	3	1

Supplementary Figure S3C All-cause death

Direct comparisons in the network

Network meta-analysis estimates	1vs4	1vs5	1vs6	2vs3	2vs4	2vs6	3vs6	4vs6
Mixed estimates								
1vs4	16.5	·	31.1	6.6	14.7	8.1	6.6	16.4
1vs5	·	100.0	·	·	·	·	·	·
1vs6	8.6	·	76.8	1.8	4.1	2.2	1.8	4.5
2vs3	0.3	·	0.3	91.6	0.9	2.8	3.6	0.5
2vs4	10.0	·	10.0	11.7	26.2	14.3	11.7	16.1
2vs6	3.5	·	3.5	24.4	9.1	29.7	24.4	5.6
3vs6	2.9	·	2.9	32.7	7.7	25.0	24.1	4.7
4vs6	13.9	·	13.9	9.1	20.3	11.1	9.1	22.5
Indirect estimates								
1vs2	6.5	·	35.2	15.2	8.0	18.5	15.2	1.5
1vs3	5.8	·	32.3	22.7	7.0	15.8	15.3	1.2
2vs5	4.6	29.4	24.8	10.7	5.6	13.1	10.7	1.1
3vs4	8.0	·	8.0	30.2	20.2	10.0	10.8	12.9
3vs5	4.2	27.6	23.4	16.5	5.0	11.4	11.1	0.8
4vs5	11.2	32.2	21.1	4.5	10.0	5.5	4.5	11.1
5vs6	4.6	46.1	41.4	1.0	2.2	1.2	1.0	2.4
**Entire network**	7.0	15.6	22.5	16.5	9.7	11.7	10.4	6.6
**Included studies**	1	1	6	2	1	1	2	1

1vs4:OAC vs Antiplatelet,1vs5:OAC vs LAAO ,1vs6:OAC vs No therapy ,2vs3NOACvsWarfarin 2vs4NOAC vs Antiplatelet ,2vs6NOAC vs No therapy ,3vs4:Warfarin vs Antiplatelet,3vs6:Warfarin vs No therapy,4vs6:Antiplatelet vs No therapy.

Supplementary Figure S4 Cumulative probability ranking plots of different intervention strategies for each outcome

Supplementary Figure S4A Cumulative probability ranking plots of Thromboembolism

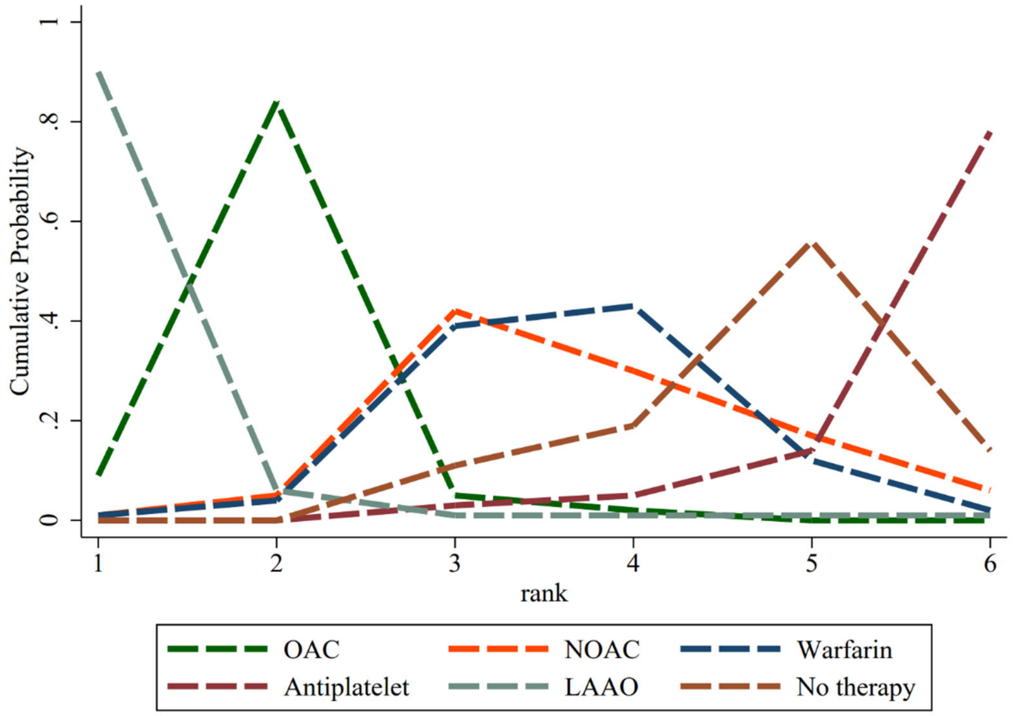

Supplementary Figure S4B Cumulative probability ranking plots of recurrent intracranial hemorrhage

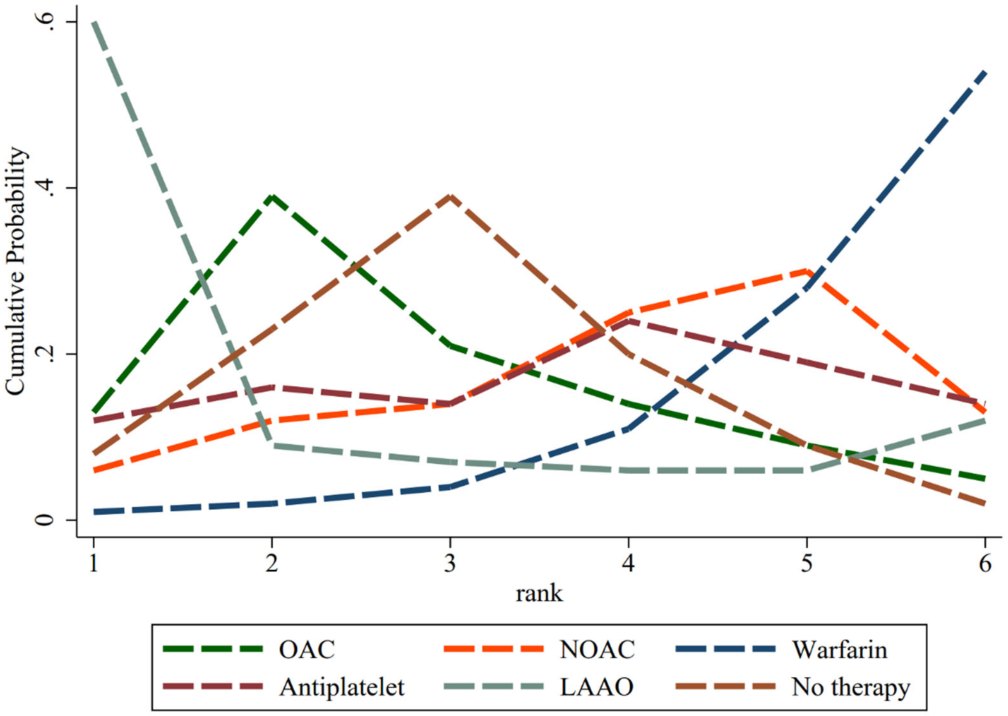

Supplementary Figure S4C Cumulative probability ranking plots of all-cause death

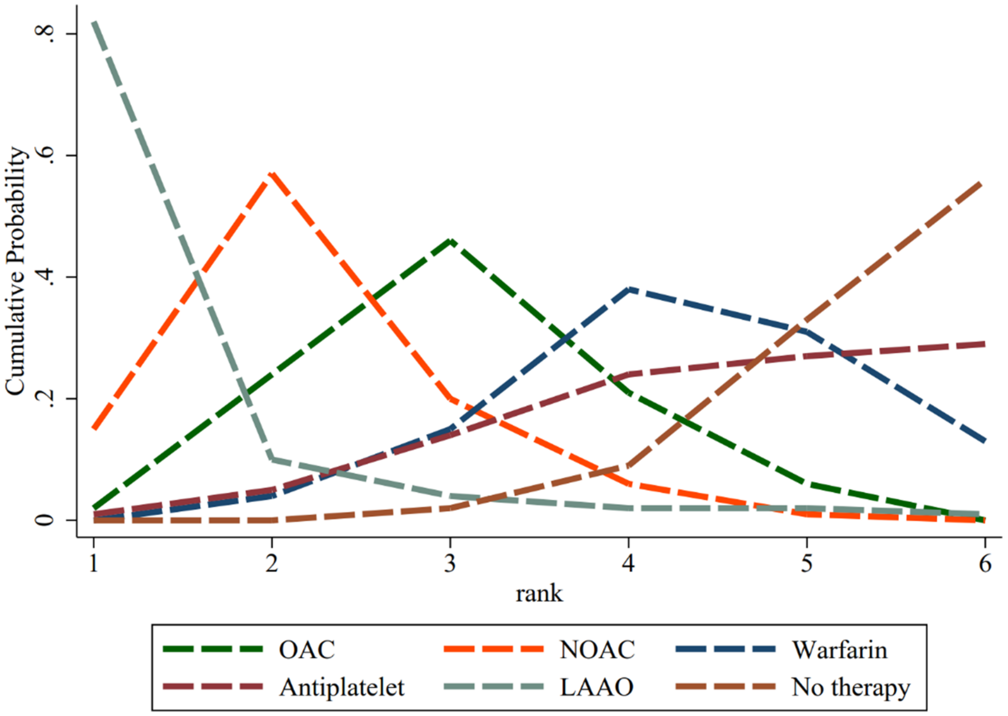

Supplementary Figure S5 Direct comparison of forest plots for each outcome

Supplementary Figure S5A Forest plot for direct comparison of thromboembolism

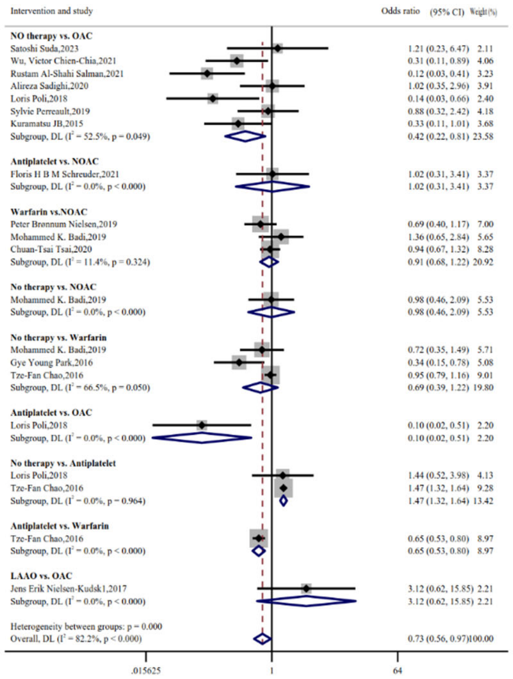

Supplementary Figure S5B Forest plot for direct comparison of recurrent intracranial hemorrhage

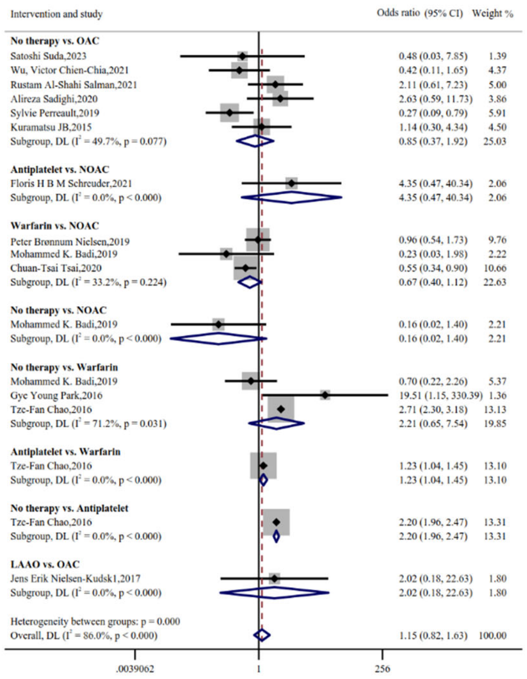

Supplementary Figure S5C Forest plot for direct comparison of all-cause death

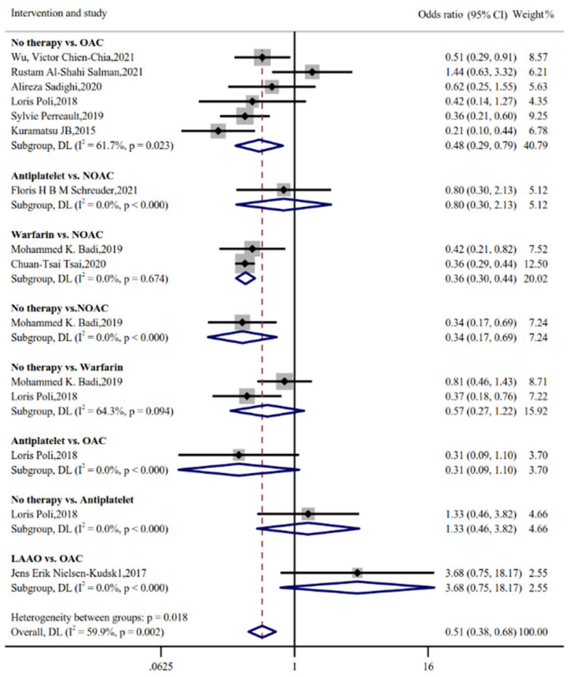

Supplementary Figure S6 Sensitivity analysis

Supplementary Figure S6A Sensitivity analysis of thromboembolism

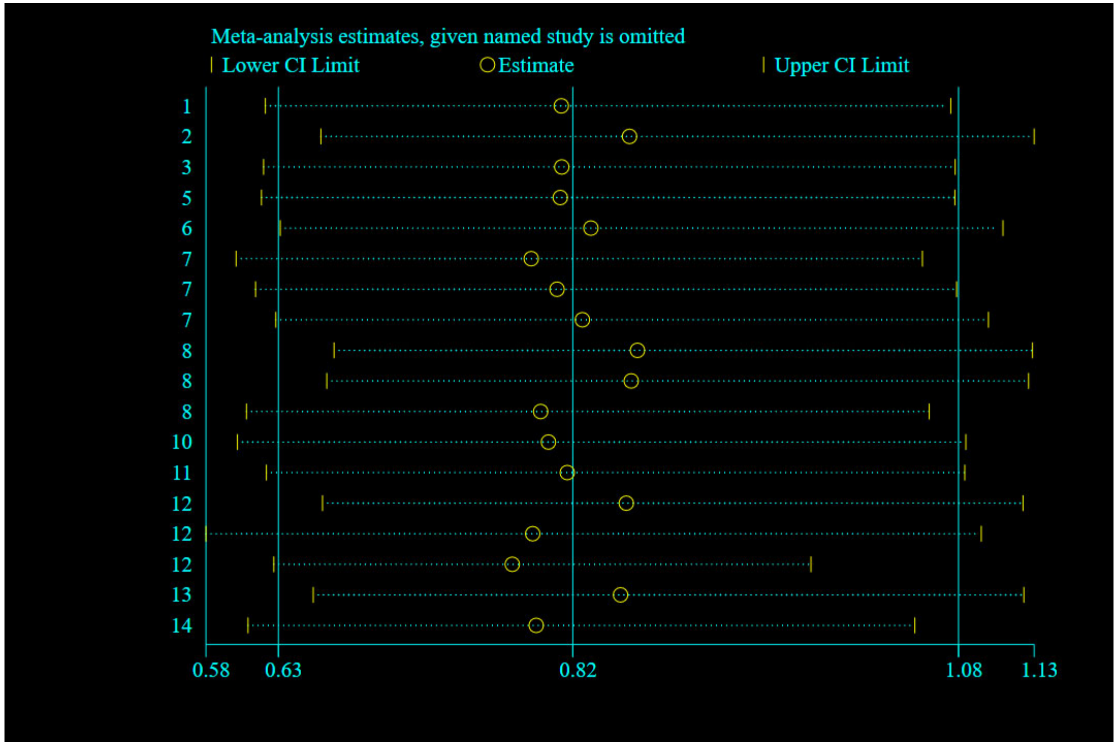

1:Satoshi Suda,2023,**2:**Wu,Victor Chien-Chia,2021,**3:**Floris H B M Schreuder,2021,**5:**Alireza Sadighi,2020,**6:**Peter Brønnum Nielsen,2019,**7:**Mohammed K. Badi,2019, **8:**Loris Poli,2018,**10:**Chuan-Tsai Tsai,2020,**11:**Sylvie Perreault,2019,**12:**Tze-Fan Chao,2016,**13:**Kuramatsu JB,2015,**14:**Jens Erik Nielsen-Kudsk1,2017

Supplementary Figure S6B Sensitivity analysis of recurrent intracranial hemorrhage

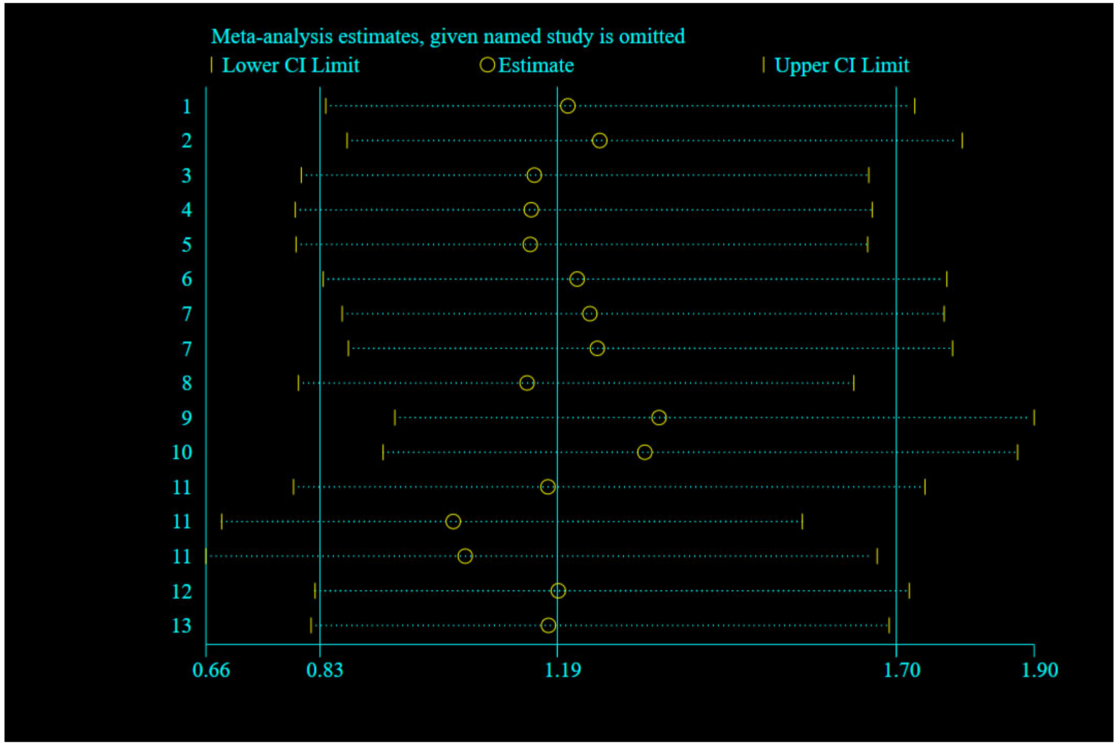

1:Satoshi Suda,2023,**2:**Wu,Victor Chien-Chia,2021,**3:**Floris H B M Schreuder,2021,**4:** Rustam Al-Shahi Salman,2021,**5:**Alireza Sadighi,2020,**6:**Peter Brønnum Nielsen, 2019,**7:**Mohammed K. Badi,2019,**8:**Gye Young Park,2016,**9:**Chuan-Tsai Tsai,2020,**10:**Sylvie Perreault,2019,**11:**Tze-Fan Chao,2016,**12:**Kuramatsu JB,2015,**13:**Jens Eri k Nielsen-Kudsk1,2017

Supplementary Figure S6C Sensitivity analysis of all-cause death

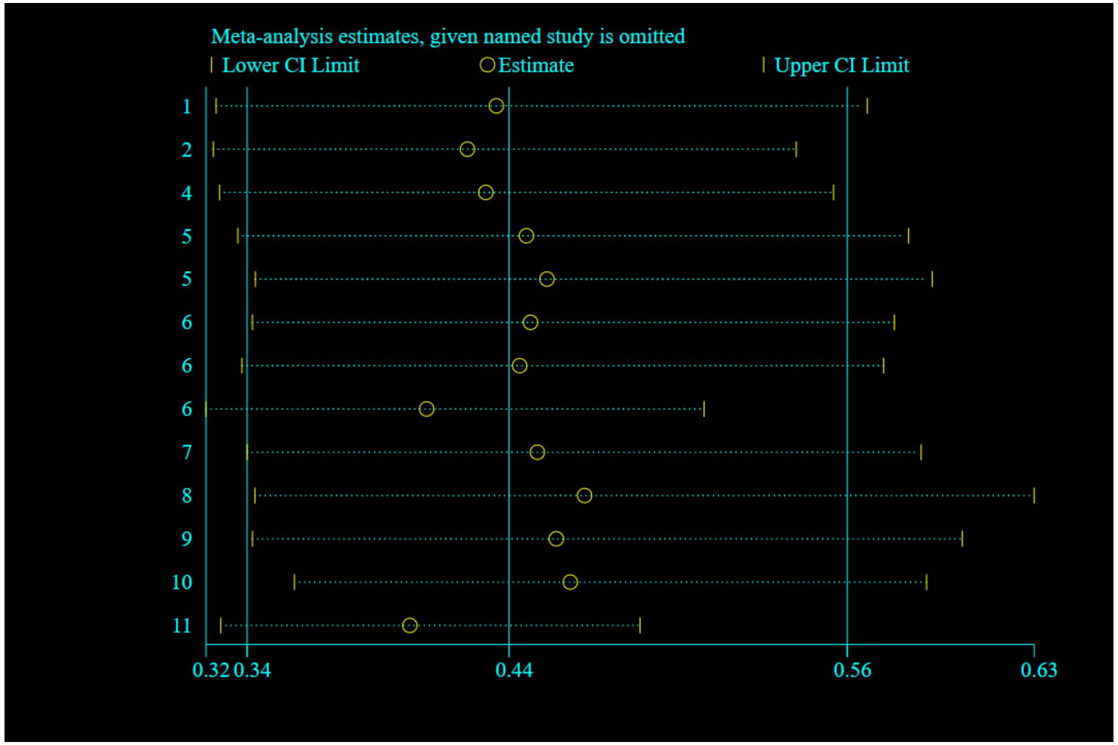

1:Wu,Victor Chien-Chia,2021,**2:**Floris H B M Schreuder,2021,**4:**Alireza Sadighi,2020,**5:**Mohammed K. Badi,2019,**6:**Loris Poli,2018,**7:**Gye Young Park,2016,**8:**Chuan-Tsai Tsai,2020,**9:**Sylvie Perreault,2019,**10:**Kuramatsu JB,2015,**11:**Jens Erik Nielsen-Kudsk1,2017.

Supplementary Methods S1. PRISMA Main Checklist

Topic	No.	Item	The location where item is reported
**TITLE**			
**Title**	1	Identify the report as a systematic review.	Pag.1
**ABSTRACT**			
**Abstract**	2	See the PRISMA 2020 for Abstracts checklist	Pag.2
**INTRODUCTION**			
**Rationale**	3	Describe the rationale for the review in the context of existing knowledge.	Pag.3
**Objectives**	4	Provide an explicit statement of the objective(s) or question(s) the review addresses.	Pag.3
**METHODS**			
**Eligibility criteria**	5	Specify the inclusion and exclusion criteria for the review and how studies were grouped for the syntheses.	Pag.3-4
**Information sources**	6	Specify all databases, registers, websites, organizations, reference lists and other sources searched or consulted to identify studies. Specify the date when each source was last searched or consulted.	Pag.4, Supplementary References
**Search strategy**	7	Present the full search strategies for all databases, registers and websites, including any filters and limits used.	Supplementary material 1 and Figure 1
**Selection process**	8	Specify the methods used to decide whether a study met the inclusion criteria of the review, including how many reviewers screened each record and each report retrieved, whether they worked independently, and if applicable, details of automation tools used in the process.	Pag.4
**Data collection process**	9	Specify the methods used to collect data from reports, including how many reviewers collected data from each report, whether they worked independently, any processes for obtaining or confirming data from study investigators, and if applicable, details of automation tools used in the process.	Pag.4
**Data items**	10a	List and define all outcomes for which data were sought. Specify whether all results that were compatible with each outcome domain in each study were sought (e.g. for all measures, time points, analyses), and if not, the methods used to decide which results to collect.	Pag.4

Topic	No.	Item	The location where item is reported
	10b	List and define all other variables for which data were sought (e.g. participant and intervention characteristics, funding sources). Describe any assumptions made about any missing or unclear information.	Pag.4, Table1
**Study risk of bias assessment**	11	Specify the methods used to assess risk of bias in the included studies, including details of the tool(s) used, how many reviewers assessed each study and whether they worked independently, and if applicable, details of automation tools used in the process.	Pag.4
**Effect measures**	12	Specify for each outcome the effect measure(s) (e.g. risk ratio, mean difference) used in the synthesis or presentation of results.	Pag. 4-5
**Synthesis methods**	13a	Describe the processes used to decide which studies were eligible for each synthesis (e.g. tabulating the study intervention characteristics and comparing against the planned groups for each synthesis (item 5)).	Pag.4
	13b	Describe any methods required to prepare the data for presentation or synthesis, such as handling of missing summary statistics, or data conversions.	Pag. 4-5
	13c	Describe any methods used to tabulate or visually display results of individual studies and syntheses.	Pag. 4-5
	13d	Describe any methods used to synthesize results and provide a rationale for the choice(s). If meta-analysis was performed, describe the model(s), method(s) to identify the presence and extent of statistical heterogeneity, and software package(s) used.	Pag. 4-5
	13e	Describe any methods used to explore possible causes of heterogeneity among study results (e.g. subgroup analysis, meta-regression).	Pag. 4-5
	13f	Describe any sensitivity analyses conducted to assess robustness of the synthesized results.	Pag. 4-5
**Reporting bias assessment**	14	Describe any methods used to assess risk of bias due to missing results in a synthesis (arising from reporting biases).	NA
**Certainty assessment**	15	Describe any methods used to assess certainty (or confidence) in the body of evidence for an outcome.	Page. 4
**RESULTS**			
**Study selection**	16a	Describe the results of the search and selection process, from the number of records identified in the search to the number of studies included in the review, ideally using a flow diagram.	Pag.5, Fig.1
	16b	Cite studies that might appear to meet the inclusion criteria, but which were excluded, and explain why they were excluded.	NA
**Study characteristics**	17	Cite each included study and present its characteristics.	Table 1, Supplementary Reference
**Risk of bias in studies**	18	Present assessments of risk of bias for each included study.	Supplementary Figure 1

Topic	No.	Item	The location where item is reported
**Results of individual studies**	19	For all outcomes, present, for each study: (a) summary statistics for each group (where appropriate) and (b) an effect estimates and its precision (e.g. confidence/credible interval), ideally using structured tables or plots.	Pag.5-6, Figure2-Figure5
**Results of syntheses**	20a	For each synthesis, briefly summarise the characteristics and risk of bias among contributing studies.	Pag.5-6
	20b	Present results of all statistical syntheses conducted. If meta-analysis was done, present for each the summary estimate and its precision (e.g. confidence/credible interval) and measures of statistical heterogeneity. If comparing groups, describe the direction of the effect.	Pag. 5-6
	20c	Present results of all investigations of possible causes of heterogeneity among study results.	Pag. 6
	20d	Present results of all sensitivity analyses conducted to assess the robustness of the synthesized results.	Supplementary Figure 6
**Reporting biases**	21	Present assessments of risk of bias due to missing results (arising from reporting biases) for each synthesis assessed.	NA
**Certainty of evidence**	22	Present assessments of certainty (or confidence) in the body of evidence for each outcome assessed.	Pag. 6
**DISCUSSION**			
**Discussion**	23a	Provide a general interpretation of the results in the context of other evidence.	Pag. 7-8
	23b	Discuss any limitations of the evidence included in the review.	Pag. 7-8
	23c	Discuss any limitations of the review processes used.	Pag.8
	23d	Discuss implications of the results for practice, policy, and future research.	Pag.8-9
**OTHER INFORMATION**			
**Registration and protocol**	24a	Provide registration information for the review, including register name and registration number, or state that the review was not registered.	Pag. 3
	24b	Indicate where the review protocol can be accessed, or state that a protocol was not prepared.	Pag. 3
	24c	Describe and explain any amendments to information provided at registration or in the protocol.	Pag.3

Topic	No.	Item	The location where item is reported
**Support**	25	Describe sources of financial or non-financial support for the review, and the role of the funders or sponsors in the review.	Pag.9
**Competing interests**	26	Declare any competing interests of review authors.	Pag.9
**Availability of data, code and other materials**	27	Report which of the following are publicly available and where they can be found: template data collection forms; data extracted from included studies; data used for all analyses; analytic code; any other materials used in the review.	Pag.9

Supplementary Methods S2. PRISMA Abstract Checklist

Topic	No.	Item	Reported?
**TITLE**			
**Title**	1	Identify the report as a systematic review.	Yes
**BACKGROUND**			
**Objectives**	2	Provide an explicit statement of the main objective(s) or question(s) the review addresses.	Yes
**METHODS**			
**Eligibility criteria**	3	Specify the inclusion and exclusion criteria for the review.	Yes
**Information sources**	4	Specify the information sources (e.g. databases, registers) used to identify studies and the date when each was last searched.	Yes
**Risk of bias**	5	Specify the methods used to assess risk of bias in the included studies.	Yes
**Synthesis of results**	6	Specify the methods used to present and synthesize results.	Yes
**RESULTS**			
**Included studies**	7	Give the total number of included studies and participants and summarise relevant characteristics of studies.	Yes
**Synthesis of results**	8	Present results for main outcomes, preferably indicating the number of included studies and participants for each. If meta-analysis was done, report the summary estimate and confidence/credible interval. If comparing groups, indicate the direction of the effect (i.e. which group is favoured).	Yes
**DISCUSSION**			
**Limitations of evidence**	9	Provide a brief summary of the limitations of the evidence included in the review (e.g. study risk of bias, inconsistency and imprecision).	Yes
**Interpretation**	10	Provide a general interpretation of the results and important implications.	Yes
**OTHER**			
**Funding**	11	Specify the primary source of funding for the review.	Yes
**Registration**	12	Provide the register name and registration number.	Yes

References

1. Schreuder F, van Nieuwenhuizen KM, Hofmeijer J, Vermeer SE, Kerkhoff H, Zock E, Luijckx GJ, Messchendorp GP, van Tuijl J, Bienfait HP, et al. Apixaban versus no anticoagulation after anticoagulation-associated intracerebral haemorrhage in patients with atrial fibrillation in the Netherlands (APACHE-AF): a randomised, open-label, phase 2 trial. The lancet Neurology. 2021;20(11):907‐16.

2. Al-Shahi Salman R, Keerie C, Stephen J, Lewis S, Norrie J, Dennis MS, Newby DE, Wardlaw JM, Lip GYH, Parry-Jones A, et al. Effects of oral anticoagulation for atrial fibrillation after spontaneous intracranial haemorrhage in the UK: a randomised, open-label, assessor-masked, pilot-phase, non-inferiority trial. The Lancet Neurology. 2021;20(10):842-53.

3. Suda S, Iguchi Y, Yagita Y, Kanzawa T, Okubo S, Fujimoto S, Kono Y, Kimura K. Resumption of oral anticoagulation in patients with non-valvular atrial fibrillation after intracerebral hemorrhage: A sub-analysis of the PASTA registry study. Journal of the Neurological Sciences. 2023;453.

4. Wu VC, Huang YC, Chen SW, Liu CH, Chang CW, Chen CC, Chang SH, Lin MS, Lee TH, Chen MC, et al. Resuming anticoagulation in patients with atrial fibrillation experiencing intracranial hemorrhage. Medicine (Baltimore). 2021;100(32):e26945.

5. Sadighi A, Wasko L, DiCristina H, Wagner T, Wright K, Capone K, Monczewski M, Kester M, Bourdages G, Griessenauer C, et al. Long-term outcome of resuming anticoagulation after anticoagulation-associated intracerebral hemorrhage. eNeurologicalSci. 2020;18.

6. Nielsen PB, Skjøth F, Søgaard M, Kjældgaard JN, Lip GYH, Larsen TB. Non-Vitamin K Antagonist Oral Anticoagulants Versus Warfarin in Atrial Fibrillation Patients With Intracerebral Hemorrhage. Stroke. 2019;50(4):939-46.

7. Badi MK, Vilanilam GK, Gupta V, Barrett KM, Lesser ER, Cochuyt JJ, Hodge DO, Brott TG, Meschia JF. Pharmacotherapy for Patients with Atrial Fibrillation and Cerebral Microbleeds. J Stroke Cerebrovasc Dis. 2019;28(8):2159-67.

8. Poli L, Grassi M, Zedde M, Marcheselli S, Silvestrelli G, Sessa M, Zini A, Paciaroni M, Azzini C, Gamba M, et al. Anticoagulants Resumption after Warfarin-Related Intracerebral Haemorrhage: The Multicenter Study on Cerebral Hemorrhage in Italy (MUCH-Italy). Thromb Haemost. 2018;118(3):572-80.

9. Nielsen PB, Larsen TB, Skjøth F, Lip GY. Outcomes Associated With Resuming Warfarin Treatment After Hemorrhagic Stroke or Traumatic Intracranial Hemorrhage in Patients With Atrial Fibrillation. JAMA Intern Med. 2017;177(4):563-70.

10. Park YA, Uhm JS, Pak HN, Lee MH, Joung B. Anticoagulation therapy in atrial fibrillation after intracranial hemorrhage. Heart Rhythm. 2016;13(9):1794-802.

11. Tsai C-T, Liao J-N, Chiang C-E, Lin Y-J, Chang S-L, Lo L-W, Hu Y-F, Tuan T-C, Chung F-P, Chao T-F, et al. Association of Ischemic Stroke, Major Bleeding, and Other Adverse Events With Warfarin Use vs Non-vitamin K Antagonist Oral Anticoagulant Use in Patients With Atrial Fibrillation With a History of Intracranial Hemorrhage. JAMA network open. 2020;3(6):e206424.

12. Lee S-R, Choi E-K, Kwon S, Jung J-H, Han K-D, Cha M-J, Oh S, Lip GYH. Oral Anticoagulation in Asian Patients With Atrial Fibrillation and a History of Intracranial Hemorrhage. Stroke. 2020;51(2):416-23.

13. Perreault S, Côté R, White-Guay B, Dorais M, Oussaïd E, Schnitzer ME. Anticoagulants in Older Patients with Nonvalvular Atrial Fibrillation after Intracranial Hemorrhage. Journal of Stroke. 2019;21(2):195-206.

14. Chao T-F, Liu C-J, Liao J-N, Wang K-L, Lin Y-J, Chang S-L, Lo L-W, Hu Y-F, Tuan T-C, Chung F-P, et al. Use of Oral Anticoagulants for Stroke Prevention in Patients With Atrial Fibrillation Who Have a History of Intracranial Hemorrhage. Circulation. 2016;133(16):1540-7.

15. Kuramatsu JB, Gerner ST, Schellinger PD, Glahn J, Endres M, Sobesky J, Flechsenhar J, Neugebauer H, Jüttler E, Grau A, et al. Anticoagulant reversal, blood pressure levels, and anticoagulant resumption in patients with anticoagulation-related intracerebral hemorrhage. JAMA. 2015;313(8):824-36.

16. Nielsen-Kudsk JE, Johnsen SP, Wester P, Damgaard D, Airaksinen J, Lund J, De Backer O, Pakarinen S, Odenstedt J, Vikman S, et al. Left atrial appendage occlusion versus standard medical care in patients with atrial fibrillation and intracerebral haemorrhage: a propensity score-matched follow-up study. EuroIntervention: Journal of EuroPCR in Collaboration with the Working Group on Interventional Cardiology of the European Society of Cardiology. 2017;13(3):371-8.
