# Supplementary material for: Efficacy and Safety of Drug and Device Strategies for Stroke Prevention in Atrial Fibrillation After Intracranial Hemorrhage: A Bayesian Network Meta-Analysis
Source: J Cardiovasc Dev Dis. 2025 Nov 28;12(12):464. doi: 10.3390/jcdd12120464 (PMC12733742; doi:10.3390/jcdd12120464)
Supplement: Supplementary file 1 [file jcdd-12-00464-s001.zip › Supplementary material S3.pdf]

ClinicalTrials.gov Search Results 02/01/2025

	Title	Status	Study Results	Conditions	Interventions	Locations
1	Comparison of LAA-Closure vs Oral Anticoagulation in Patients With NVAF and Status Post Intracranial Bleeding.	Recruiting	No Results Available	• Atrial Fibrillation (AF)<br>• Intracranial Bleed	• Device: Percutaneous closure of the LAA (Watchman / Watchman FLX)	• Universitätsherzzentrum Freiburg - Bad Krozingen, Freiburg, Baden Württemberg, Germany<br>• Klinikum Friedrichshafen GmbH, Friedrichshafen, Baden-Wurttemberg, Germany<br>• Universitätsklinikum der J.W. Goethe-Universität Frankfurt, Frankfurt am main, Hessen, Germany<br>• Knappschaftskrankenhaus Bottrop Gmbh, Bottrop, Nordrhein Westfahlen, Germany<br>• Klinikum Chemnitz, Chemnitz, Sachsen, Germany<br>• Städtisches Klinikum Friedrichstadt Dresden, Dresden, Sachsen, Germany<br>• Klinikum St. Georg gGmbH, Leipzig, Sachsen, Germany<br>• HBK Zwickau, Zwickau, Sachsen, Germany<br>• Katholisches Krankenhaus "St. Johann Nepomuk", Erfurt, Thüringen, Germany<br>• SRH Wald-Klinikum Gera GmbH, Gera, Thüringen, Germany<br>• and 32 more
2	Prevention of Stroke by Left Atrial Appendage Closure in Atrial Fibrillation Patients After Intracerebral Hemorrhage	Recruiting	No Results Available	• Atrial Fibrillation<br>• Stroke<br>• Intracerebral Hemorrhage	• Device: LAAO<br>• Drug: Medical Therapy	• Aarhus University Hospital, Aarhus, Denmark<br>• Bispebjerg University Hospital, Bispebjerg, Denmark<br>• Rikshospitalet Glostrup, Glostrup, Denmark<br>• Herlev sjukhus, Herlev, Denmark<br>• Odenses Universitetssjukhus, Odense, Denmark<br>• Helsinki University Hospital, Helsinki, Finland<br>• North Karelia Central Hospital, Joensuu, Finland<br>• Kuopio University Hospital, Kuopio, Finland<br>• Turku University Hospital, Turku, Finland<br>• Vaasa Centralsjukhus, Vaasa, Finland<br>• and 7 more
